# Mindfulness and anxiety among young family caregivers: the mediating roles of psychological flexibility and self-compassion

**DOI:** 10.3389/fpsyg.2026.1876251

**Published:** 2026-10-23

**Authors:** Yunfu Liu, Shuai Zhong, Cheng Yang

**Affiliations:** 1 School of Physical Education and Health Sciences, Guangxi Science & Technology Normal University, Laibin, Guangxi, China; 2 College of Innovation and Management, Suan Sunandha Rajabhat University, Bangkok, Thailand

**Keywords:** anxiety, mindfulness, psychological flexibility, self-compassion, young family caregivers

## Abstract

**Introduction:**

During critical developmental periods, young family caregivers frequently take on unpaid caregiving for family members who are physically ill, have disabilities, or have mental health conditions. Ongoing caregiving pressure may be associated with a greater risk of anxiety and poorer mental health. Psychological flexibility and self-compassion reflect distinct but interrelated psychological processes involved in responding to stress and may help characterize the association between mindfulness and anxiety. Accordingly, among young family caregivers, this study examined the association between mindfulness and anxiety and whether psychological flexibility and self-compassion function as sequential mediators.

**Methods:**

A total of 485 young family caregivers aged 15–24 were recruited from three prefecture-level cities in Hunan Province, China. All participants completed measures of mindfulness, psychological flexibility, self-compassion, and anxiety. Structural equation modeling was used to examine the hypothesized relationships, and the serial indirect effect was examined using 5,000 bootstrap resamples with bias-corrected 95% confidence intervals.

**Results:**

Higher mindfulness was associated with greater psychological flexibility and self-compassion and lower anxiety; greater psychological flexibility was associated with greater self-compassion and lower anxiety, while greater self-compassion was associated with lower anxiety. The hypothesized serial indirect association from mindfulness to anxiety through psychological flexibility and self-compassion was significant (*β* = −0.128, 95% CI [−0.167, −0.096]).

**Discussion:**

The findings support the relevance of the stress process model to young family caregiving and suggest that anxiety is associated not only with caregiving burden, but also with how caregiving-related stress is processed and how individuals respond to their own distress. These findings point to the potential value of considering internal psychological resources alongside the family caregiving context and support from schools, communities, and service systems.

## 1 Introduction

Adolescence and young adulthood are critical developmental periods during which individuals pursue education, explore career pathways, develop social relationships, and gradually attain independence (Arnett, 2000). However, some young people also undertake regular, unpaid caregiving responsibilities for family members with physical illnesses, disabilities, mental health conditions, or substance-use problems. These individuals are often referred to as young family caregivers or young carers (Chevrier et al., 2022). Compared with peers without caregiving responsibilities, young family caregivers may need to manage long-term care demands while also navigating academic, occupational, and social developmental tasks. This overlap may increase chronic stress, role imbalance, and psychological vulnerability (Darabos and Faust, 2023; King et al., 2025). Anxiety deserves particular attention in this context because the uncertainty of long-term caregiving, together with constraints on education, employment, and social participation, may intensify worries about current circumstances and future opportunities (Fleitas Alfonzo et al., 2022). Previous research indicates that youth carers often experience poorer mental health, educational adjustment, and social functioning than non-caregiving peers, and these difficulties may become more pronounced as caregiving intensity and duration increase (Lacey et al., 2025; Lacey et al., 2022). Moreover, because young people’s caregiving roles are often hidden within the family context, their psychological risks may be difficult to identify and address in a timely manner (Masterson-Algar et al., 2023). Therefore, anxiety among young family caregivers should be understood not merely as a general emotional problem, but as a mental health concern embedded in a sensitive developmental and caregiving context.

Mindfulness has been widely examined as a psychological resource associated with lower anxiety. It is commonly defined as an open, receptive, and non-judgmental awareness of present-moment experiences (Brown and Ryan, 2003). Higher levels of mindfulness may reduce anxiety by weakening automatic involvement in threatening thoughts, decreasing worry and rumination, and improving emotion regulation (Carpenter et al., 2019; Roemer et al., 2015). Previous studies have shown that mindfulness is negatively associated with cognitive and emotional distress and that mindfulness-based interventions can reduce anxiety symptoms by altering the processing of negative emotions (Desrosiers et al., 2013; Haller et al., 2021). In caregiver populations, mindfulness-based interventions have also been found to reduce anxiety, stress, and caregiving burden while improving psychological well-being (Zhang et al., 2025). For young family caregivers, mindfulness may be particularly relevant because it does not necessarily remove caregiving demands, but may influence how young people attend to, interpret, and respond to caregiving-related stress.

Although the inverse association between mindfulness and anxiety has been documented in previous research, the mechanisms underlying this association remain insufficiently understood among young family caregivers. Existing studies have often explained the protective role of mindfulness through worry, rumination, and general emotion regulation (Desrosiers et al., 2013; Roemer et al., 2015). Less attention has been paid to how mindfulness may operate through adaptive psychological resources that are especially relevant under long-term caregiving stress. Psychological flexibility refers to the ability to remain open to present-moment experiences and to persist in value-consistent actions despite stress, discomfort, or adversity (Kashdan and Rottenberg, 2010). Self-compassion refers to responding to one’s own suffering with self-kindness, a sense of common humanity, and balanced awareness rather than self-criticism or over-identification (Neff, 2003). These two resources may be important for young family caregivers because they often face not only caregiving demands, but also developmental challenges related to education, employment, social relationships, and role formation (Fleitas Alfonzo et al., 2022). Compared with peers without caregiving responsibilities, young caregivers may be more likely to experience role conflict, elevated self-imposed expectations, social isolation, and reduced psychological well-being (Brimblecombe et al., 2024; Fingerman et al., 2025; Haugland et al., 2022). However, research on psychological flexibility and self-compassion has focused mainly on professional caregivers or adult family caregivers, whereas evidence among young family caregivers remains limited (Jansen et al., 2017; Murfield et al., 2024). Therefore, examining these mechanisms in young family caregivers may help clarify how mindfulness is linked to anxiety in this under-recognized population.

The stress process model provides a useful theoretical framework for understanding anxiety among caregivers. According to this model, caregivers’ psychological distress is associated not only with caregiving-related stressors, but also with coping processes and the personal and social resources available to them (Pearlin et al., 1990). The stress process may be particularly salient for young family caregivers because caregiving responsibilities often occur alongside age-related developmental demands, including schooling, employment preparation, identity exploration, and social participation (Pope et al., 2022). From this perspective, anxiety may reflect not only exposure to caregiving-related stress, but also the ways in which young caregivers process stress and mobilize internal psychological resources. Within this framework, mindfulness, psychological flexibility, and self-compassion can be conceptualized as distinct but related psychological resources and processes, whereas anxiety represents the psychological outcome of interest. Mindfulness can be viewed as a personal psychological resource, psychological flexibility as an adaptive process involved in responding to difficult internal experiences, and self-compassion as a self-directed regulatory resource reflecting how individuals relate to themselves under distress. More specifically, mindfulness is characterized by open, receptive, and non-judgmental awareness of present-moment internal and external experiences (Bishop et al., 2004). This awareness may reduce automatic negative reactions to stress and facilitate greater psychological flexibility (Böge et al., 2023; Brown et al., 2013). Psychological flexibility reflects the capacity to remain open to difficult internal experiences and to respond adaptively rather than through rigid avoidance or defensive reactions. Such openness may reduce experiential avoidance and rigid self-evaluation, making it easier for individuals to acknowledge distress and personal limitations without becoming dominated by self-criticism. This may, in turn, create more favorable conditions for self-compassion, which involves responding to one’s own suffering with kindness, acceptance, and balanced awareness (Marshall and Brockman, 2016; Yadavaia et al., 2014). Higher self-compassion may also be associated with more adaptive emotional responses to stressful experiences (Leary et al., 2007) and lower levels of anxiety (Egan et al., 2022). Taken together, this framework provides a theoretical basis for examining the association between mindfulness and anxiety and the proposed indirect associations involving psychological flexibility and self-compassion among young family caregivers.

Despite growing evidence linking mindfulness, psychological flexibility, self-compassion, and anxiety, their interrelationships remain insufficiently understood among young family caregivers. In particular, limited research has examined whether psychological flexibility and self-compassion jointly characterize the indirect association between mindfulness and anxiety in this population, or whether their proposed sequential relationship is evident within this association. Addressing this gap is particularly relevant for young family caregivers because caregiving responsibilities often coincide with developmental demands related to education, employment, identity formation, and social participation. Guided by the stress process model, the present study aimed to: (1) examine the associations among mindfulness, psychological flexibility, self-compassion, and anxiety among young family caregivers; and (2) examine the proposed indirect association between mindfulness and anxiety involving psychological flexibility and self-compassion, including their hypothesized sequential relationship.

## 2 Research hypotheses

### 2.1 Mindfulness, psychological flexibility, and self-compassion

Individuals constantly navigate dynamic and changing environments in which they encounter not only positive experiences and opportunities for growth, but also ongoing stress, burdens, and uncertainties. For young family caregivers, such stress may be especially pronounced. Existing research indicates that adolescent caregivers aged 15–21 report poorer well-being and greater perceived stress than their non-caregiving peers (Wepf and Leu, 2022). At the same time, longitudinal studies of adult caregivers have shown that younger individuals tend to experience poorer mental health in the context of informal caregiving, and that the impact on mental health becomes more pronounced as caregiving intensity increases (Fleitas Alfonzo et al., 2024). Moreover, because this group is at a critical stage of education, employment, and personal development, family caregiving responsibilities may further exacerbate their psychological stress and life burdens (Xue et al., 2024). Mindfulness is widely regarded as an important psychological resource for promoting individual well-being and resilience. It enables individuals to direct their attention steadily to present-moment experiences, rather than becoming readily drawn into negative judgments, ruminative thinking, or emotional escalation (Davis et al., 2024). Although mindfulness does not directly reduce objective stress, it may reshape the way individuals respond to stressful experiences, thereby laying the foundation for more effective self-regulation (Toniolo-Barrios and ten Brummelhuis, 2023).

Masuda and Tully (2012) noted that although mindfulness and psychological flexibility are distinct concepts, they are closely related in terms of function. Kashdan and Rottenberg (2010) further suggested that psychological flexibility is better understood not as a single trait, but as a set of dynamic processes organized around the capacity to recognize and adjust to situational demands, shift psychological resources and behavioral strategies when existing approaches are no longer effective, and maintain a balance across competing needs, goals, and life domains. In Acceptance and Commitment Therapy (ACT), psychological flexibility is conceptualized as a central therapeutic target. This perspective emphasizes that individuals should fully engage with their present experiences while reducing avoidance and rigid responses, and persist in or modify their behaviors in accordance with personally valued goals (Hayes et al., 2006). Based on this logic, the mindfulness practices of present-moment awareness and non-judgmental acceptance may help reduce individuals’ automatic, defensive, and avoidant responses in stressful situations, enhance their openness to contextual information, and thereby broaden their range of behavioral options and capacity for value-driven action. Feng et al. (2025) found psychological flexibility to mediate the influence of mindfulness on college students’ mental health. Tan et al. (2023) found, among family caregivers of people with dementia, that higher levels of psychological flexibility corresponded to less caregiving burden.

Self-compassion likewise serves as a key marker of positive psychological adjustment. Neff (2003) defines self-compassion as an individual’s capacity to respond to suffering, failure, or personal shortcomings with kindness, understanding, and acceptance, rather than with excessive self-criticism, isolation, or over-identification with negative experiences. At its core, self-compassion does not involve denying problems or turning away from reality; it means relating to oneself with greater balance and kindness when facing hardship (Neff, 2023). For young family caregivers, self-compassion entails maintaining self-understanding and self-acceptance under caregiving pressure, while resisting excessive self-criticism and preserving the capacity to care for themselves amid overlapping role conflicts, caregiving demands, and developmental challenges. Consistent with this, mindfulness has also been linked to higher self-compassion (Golden et al., 2021). Mindfulness enables individuals to perceive painful experiences in a more balanced manner, thereby reducing excessive involvement in negative emotions and self-criticism and laying the foundation for responding to oneself with kindness and acceptance (Biehler and Naragon-Gainey, 2022). In other words, when individuals are no longer entangled in the automatic evaluations and emotional reactions elicited by stressful situations, they are more likely to respond to frustration, helplessness, and exhaustion with self-understanding rather than self-criticism.

Although psychological flexibility and self-compassion are distinct constructs, they are conceptually and empirically related. Psychological flexibility centers on adjusting behavior to situational demands and maintaining engagement in meaningful activities despite negative experiences (Qi et al., 2025). Self-compassion centers on responding to personal suffering and limitations with acceptance, understanding, and kindness (Mey et al., 2023). Although these two constructs emphasize adaptive responding and self-directed attitudes, respectively, both involve a more open and less judgmental orientation toward difficult experiences. Marshall and Brockman (2016) reported that both psychological flexibility and self-compassion were significantly associated with better emotional health. Davey et al. (2020) reported that emotional resilience and self-compassion, although interrelated, represented relatively distinct constructs, and that each was linked to better daily functioning and lower levels of depression. From the perspective of the psychological flexibility model, greater openness to difficult internal experiences and reduced experiential avoidance may allow individuals to disengage from rigid self-evaluative responses and relate to their distress with greater acceptance (Hayes et al., 2006; Marshall and Brockman, 2016). This proposed direction is also supported by intervention evidence: Yadavaia et al. (2014) found that psychological flexibility significantly mediated changes in self-compassion following an Acceptance and Commitment Therapy intervention. Based on this logic, individuals with greater psychological flexibility tend to approach painful experiences with greater openness, rather than responding to negative experiences through resistance, avoidance, or rigid control. At the same time, they are better able to adjust their cognitive and behavioral patterns in accordance with situational demands, thereby reducing unnecessary psychological strain and self-doubt. For young family caregivers, greater psychological flexibility may make individuals more willing to accept their own imperfections and emotional fluctuations under ongoing caregiving stress. They are better able to avoid excessive self-blame, shame, and helplessness arising from caregiving challenges, and instead respond to themselves with greater kindness, understanding, and support.

Based on the aforementioned theories and empirical findings, it can be inferred that mindfulness may enhance psychological flexibility among young family caregivers by reducing their automatic engagement with stress, thereby further promoting self-compassion. At the same time, greater psychological flexibility may also promote self-compassion. On this basis, the study advances the following hypotheses:

*H1*: Mindfulness is positively associated with psychological flexibility among young family caregivers.

*H2*: Mindfulness is positively associated with self-compassion among young family caregivers.

*H3*: Psychological flexibility is positively associated with self-compassion among young family caregivers.

### 2.2 Psychological flexibility, self-compassion, and anxiety

Anxiety refers to a negative emotional response that arises when individuals are confronted with threat, stress, and uncertainty, and it is typically characterized by persistent worry, tension, and excessive anticipation of potential adverse outcomes (Grupe and Nitschke, 2013). For young family caregivers, anxiety represents a mental health risk that warrants particular attention. Previous research has indicated that young family caregivers face an elevated risk of mental health problems (Lacey et al., 2022). Among young people, individuals who assume family caregiving duties have likewise been found to report greater anxiety and poorer psychological well-being (Greene et al., 2017). Young family caregivers are often required to manage continuing caregiving responsibilities while moving through pivotal stages of academic, occupational, social, and personal development. When caregiving demands intersect with these developmental tasks, they may face persistent role conflict, limited time, uncertainty about the future, and sustained emotional strain, all of which can heighten anxiety (Lacey et al., 2023; Landi et al., 2022; Xue et al., 2023). The stress process model further suggests that such stressors do not translate into psychological problems in any simple or automatic way. Instead, whether stress develops into anxiety depends on how individuals appraise and cope with these demands, as well as on the protective resources available to them (Pearlin et al., 1990). In other words, differences in anxiety among young family caregivers facing similar caregiving demands may reflect not only variation in objective stress exposure, but also differences in how stress is processed and in the internal resources available for regulation.

Psychological flexibility may alleviate anxiety by altering the way individuals process stress. According to the stress process model, when individuals are exposed to persistent stressors over an extended period, stress is more likely to manifest as chronic worry and anxiety if they tend to fixate rigidly on threatening information, avoid negative experiences, or have difficulty adjusting their cognitive and behavioral responses in a timely manner (Pearlin et al., 1990). For young caregivers, the caregiving process is often accompanied by fatigue, guilt, helplessness, and uncertainty (Xue and Guo, 2026). If they continue to be driven by these internal experiences and respond through experiential avoidance, overcontrol, or catastrophizing, they may be more likely to maintain elevated levels of anxiety (Sahib et al., 2024). Conversely, higher levels of psychological flexibility enable individuals to remain aware of their present-moment experiences even under stress and to adjust their cognition and behavior in accordance with situational demands, rather than becoming constrained by a single emotion, threatening cues, or rigid patterns of thinking (Machulska and Klucken, 2025; Westhoff et al., 2024). In other words, psychological flexibility does not eliminate stress itself, but it may alter the way individuals respond to stress by reducing excessive vigilance toward uncertainty, minimizing avoidance and amplification of negative experiences, and enabling them to maintain contact with reality and remain engaged in important life domains even under difficult circumstances. For family caregivers, the transition from stress to anxiety may be weakened when psychological flexibility is higher (Rickardsson et al., 2022).

Unlike psychological flexibility, which operates primarily through stress-processing mechanisms, self-compassion mainly influences how individuals perceive and relate to themselves in stressful situations (Biehler and Naragon-Gainey, 2022). For young family caregivers, the responsibility of providing ongoing care is often accompanied by high levels of internalized self-expectations, such as dissatisfaction with their caregiving performance and negative attributions concerning constraints on personal development (Subramaniam and Mehta, 2024). When caregiving challenges or developmental setbacks arise, such self-expectations may readily give rise to self-blame, shame, and feelings of helplessness, thereby exacerbating anxiety (Xue and Guo, 2026). By contrast, self-compassion means approaching one’s pain, setbacks, and limitations with understanding, kindness, and acceptance. This balanced self-response may reduce feelings of shame and failure in stressful situations, lessen the impact of negative self-evaluation on anxiety, and support emotional stability under adversity (Asselmann et al., 2024; Wang et al., 2024).

It is worth mentioning that, although the caregiving responsibilities, family obligations, and actual pressures faced by young family caregivers are mostly objective conditions that cannot be easily changed, anxiety is highly subjective. Simply put, two young family caregivers facing similar caregiving demands may experience different levels of anxiety due to differences in their ability to cope with stress and the coping strategies they use (Beck and Clark, 1997). Although psychological flexibility and self-compassion cannot eliminate the objective stressors encountered by these caregivers, they can still mitigate the psychological impact of these stressors through other mechanisms. Psychological flexibility seems to mainly work by reducing cognitive rigidity, experiential avoidance, and catastrophic thinking, while self-compassion may alleviate anxiety by reducing self-blame, shame, and emotional exhaustion.

Based on the aforementioned theories and empirical findings, anxiety among young family caregivers is influenced not only by the stress associated with long-term caregiving, but also by how they perceive, process, and respond to such stressful experiences. Therefore, psychological flexibility and self-compassion may protect young family caregivers from anxiety. On this basis, the study advances the following hypotheses:

*H4*: Psychological flexibility is negatively associated with anxiety among young family caregivers.

*H5*: Self-compassion is negatively associated with anxiety among young family caregivers.

### 2.3 Mediating effect

Previous research has shown that the effects of mindfulness on anxiety, depression, and stress are not always immediate, but are often achieved gradually through a series of internal psychological regulatory processes. Duarte and Pinto-Gouveia (2017) suggested that mindfulness-based interventions may work by increasing self-compassion and psychological flexibility and by decreasing psychological rigidity. In caregiver samples, mindfulness and psychological flexibility have also been related to lower anxiety symptoms (Tan et al., 2023).

Based on the stress process model, the effects of stressors on psychological outcomes are not linear but rather unfold gradually through mediating processes such as perception, appraisal, and regulation (Pearlin et al., 1990). For young family caregivers, although caregiving stress is objectively present, whether it develops into chronic anxiety depends largely on how individuals process these stressful experiences and how they relate to themselves in stressful situations. Research suggests that mindfulness is related to psychological flexibility and self-compassion, both of which have also been associated with anxiety (Davis et al., 2024; Feng et al., 2025; Hemi et al., 2024; Liang et al., 2022; Wong et al., 2025). It can be inferred that the effects of mindfulness on anxiety are unlikely to operate through a single pathway, but may instead unfold through a series of sequential psychological regulatory mechanisms. Specifically, higher levels of mindfulness may promote more open and adaptive coping by enhancing psychological flexibility among young family caregivers, thereby reducing rigid attention to threatening information, experiential avoidance, and catastrophic processing. In turn, this may enable individuals to respond to the fatigue, limitations, and emotional fluctuations arising in the caregiving process with greater understanding, acceptance, and kindness, which may in turn promote greater self-compassion. Mechanistically, the two constructs operate differently: psychological flexibility mainly shapes stress processing, while self-compassion more directly influences how individuals relate and respond to themselves. On this basis, the study advances the following hypothesis:

*H6*: Among young family caregivers, mindfulness is indirectly associated with anxiety through a sequential pathway involving psychological flexibility followed by self-compassion.

The hypothesized model is shown in Figure 1.

**Figure 1:**
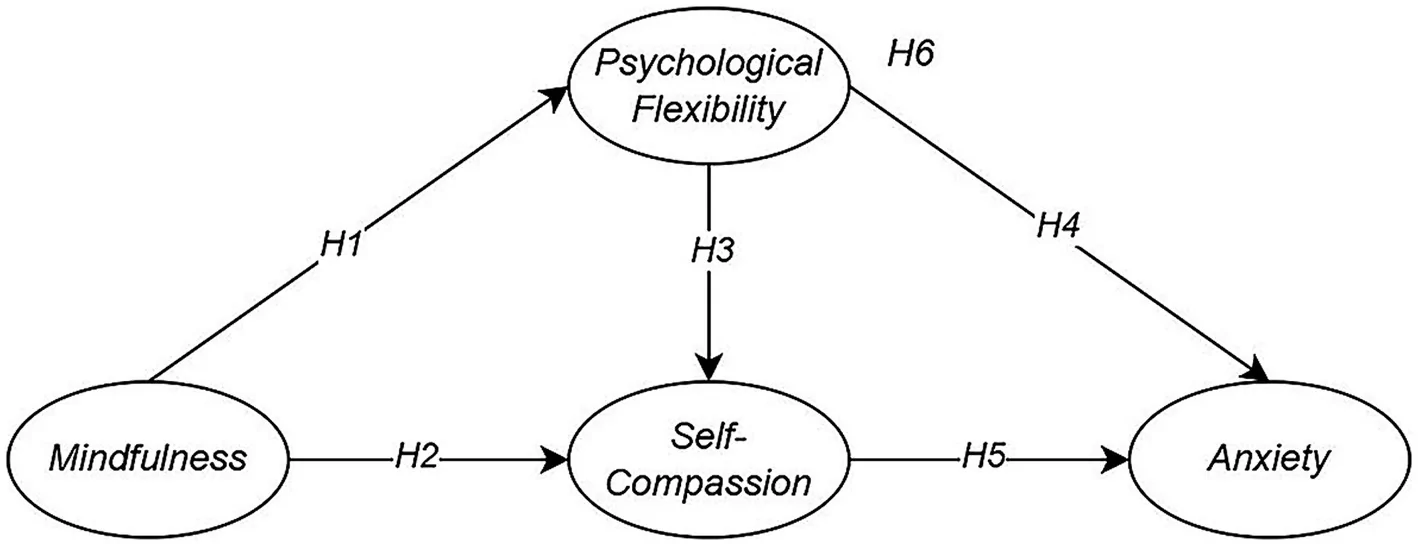
Hypothesized model.

## 3 Methods

### 3.1 Participants and procedures

According to the United Nations’ statistical definition of youth, individuals aged 15–24 years were classified as youth in this study (United Nations, n.d). Young family caregivers within this age range were recruited using convenience sampling, a non-probability sampling method. Eligibility criteria included: (1) being aged 15–24 years; (2) currently providing *regular, unpaid care or support to a family member*; (3) being able to complete the questionnaire independently; and (4) willingness to participate in the study. Exclusion criteria were: (1) professional *or paid* caregiver*s*; (2) being outside the specified age range; and (3) submitting an invalid questionnaire according to the predefined data-quality criteria described below. Between September and December 2025, face-to-face recruitment was conducted through communities and relevant service agencies in Changsha, Hengyang, and Zhuzhou, Hunan Province, China. Staff were informed of the eligibility criteria in advance and assisted in identifying and contacting potentially eligible individuals and providing basic study information. Eligible and interested individuals were then informed of the study purpose, procedures, voluntary participation, and right to withdraw before completing the questionnaire on site. The same recruitment and screening procedures were followed across all three cities. Participants who completed the questionnaire received a pack of wet wipes and a bottle of mineral water as a token of appreciation. Participants aged 15–17 years and those aged 18–24 years completed the questionnaire on site under the same conditions. Questionnaires were completed independently, with staff remaining approximately 1 m away and without viewing or interfering with responses; no directly identifying information was recorded. For participants aged 15–17 years, verbal parental or guardian consent was obtained by telephone, and participant assent was obtained separately before questionnaire completion. The study protocol was reviewed and approved by the Academic Committee of the School of Physical Education and Health Sciences, Guangxi Science & Technology Normal University, on July 10, 2025.

Prior to data collection, the minimum sample size was estimated based on a conventional participant-to-item ratio for the measurement component of the SEM. The four measures contained 57 observed items in total. Following the recommendation of at least five participants per observed variable when the total sample exceeds 100 (Streiner, 1994), the minimum target sample size was estimated to be 285. To allow for incomplete or invalid responses, the recruitment target was set above this minimum. A total of 524 questionnaires were distributed. Questionnaires were excluded if more than 20% of the items were unanswered, the total completion time was less than 3 min, or the responses showed clear non-differentiated or patterned responding, such as selecting the same response option throughout the questionnaire or following an obvious repetitive response pattern. After withdrawals and invalid questionnaires were excluded, 485 questionnaires were retained for analysis. Table 1 shows the demographic characteristics of the participants. The sample included 55.3% males and 44.7% females. In terms of age, most participants were in the 18 and under group or the 19–21 group, accounting for 49.5 and 35.7%, respectively. Students made up the largest subgroup at 54.8%. A total of 69.1% of the participants were unemployed. The distribution of places of residence was relatively balanced, with the two groups remaining close to a 1:1 ratio.

**Table 1:** Demographic characteristics (*N* = 485).

Variable	Category	*n*	%
Gender	Male	268	55.3%
	Female	217	44.7%
Age	≤18	240	49.5%
	19–21	173	35.7%
	22–24	72	14.8%
Student status	Currently enrolled	266	54.8%
	Not enrolled	219	45.2%
Employment status	Employed	150	30.9%
	Unemployed	335	69.1%
Place of residence	Urban	238	49.1%
	Rural	247	50.9%

### 3.2 Measures

Demographic information, including gender, age, student status, employment status, and place of residence, was collected in the first section. The remaining four sections assessed mindfulness, psychological flexibility, self-compassion, and anxiety in turn. All four measures were administered using established Chinese versions.

The 15-item Mindful Attention Awareness Scale (Brown and Ryan, 2003) was used. Items are rated from 1 (almost always) to 6 (almost never). Scores were averaged (range: 1–6), with higher scores indicating greater mindfulness. Previous research has demonstrated satisfactory psychometric properties of the MAAS in Chinese populations (Deng et al., 2012). Cronbach’s α was 0.952 in the present study.

The 23-item Comprehensive Assessment of Acceptance and Commitment Therapy Processes (Francis et al., 2016) was used. Items are rated from 0 (strongly disagree) to 6 (strongly agree). After reverse-scoring negatively keyed items, scores were averaged (range: 0–6), with higher scores indicating greater psychological flexibility. Previous research has provided psychometric support for this measure in Chinese populations (Zhao et al., 2024). Cronbach’s α was 0.966.

The 12-item Self-Compassion Scale–Short Form (Raes et al., 2011) was used. Items are rated from 1 (almost never) to 5 (almost always). After reverse-scoring negatively keyed items, scores were averaged (range: 1–5), with higher scores indicating greater self-compassion. Previous research has demonstrated satisfactory psychometric properties of this measure among Chinese college students (Tang et al., 2024). Cronbach’s α was 0.942.

The 7-item Generalized Anxiety Disorder Scale (Spitzer et al., 2006) was used. Items are rated from 0 (not at all) to 3 (nearly every day) based on symptoms during the previous 2 weeks. Scores were averaged (range: 0–3), with higher scores indicating greater anxiety symptoms. Previous research has demonstrated satisfactory psychometric properties of the GAD-7 among Chinese adolescents (Sun et al., 2021). Cronbach’s *α* was 0.909.

### 3.3 Data analysis

Data analysis was conducted using SPSS 27 and AMOS 24. SPSS 27 was used to obtain Pearson correlation coefficients and Cronbach’s *α* values for all variables. AMOS 24 was used to examine a structural equation model of the relationship between mindfulness and anxiety among young family caregivers. Psychological flexibility and self-compassion were included as mediating variables. Prior to SEM analysis, data screening included assessments of normality, potential multivariate outliers, multicollinearity, and missing data. Normality was examined using skewness and kurtosis statistics, with absolute skewness values below 2 and absolute kurtosis values below 7 considered acceptable (Curran et al., 1996). Potential multivariate outliers were assessed using Mahalanobis distance at *p* < 0.001 (Hair et al., 2019), and multicollinearity was examined using tolerance and variance inflation factor (VIF) values, with tolerance values above 0.20 and VIF values below 5 considered acceptable (Marcoulides and Raykov, 2019). No missing values remained in the final analytic dataset. Parameters were estimated with maximum likelihood estimation, and the analysis followed a two-step procedure (Anderson and Gerbing, 1988). A four-factor measurement model including mindfulness, psychological flexibility, self-compassion, and anxiety was first tested using CFA. All four latent constructs were estimated simultaneously, with their respective scale items specified as indicators. Model fit was evaluated using *χ*^2^/df, NFI, TLI, CFI, RMSEA, and SRMR. Acceptable fit was defined as *χ*^2^/df < 3, NFI, TLI, and CFI > 0.90, and RMSEA and SRMR < 0.08 (Hair et al., 2019). Discriminant validity was assessed using the Fornell–Larcker criterion, whereby the square root of each construct’s AVE was required to exceed its correlations with the other constructs (Fornell and Larcker, 1981). The hypothesized serial indirect effect was tested using 5,000 bootstrap resamples with bias-corrected 95% confidence intervals. Statistical significance was determined when the confidence interval did not include zero. The individual indirect effects through psychological flexibility alone or self-compassion alone were not separately tested, as the mediation analysis focused on the hypothesized serial indirect pathway. Harman’s single-factor test was used to conduct a preliminary assessment of common method bias. The first factor accounted for 22.46% of the total variance, below the commonly used threshold, indicating that common method bias was unlikely to be a serious concern (Podsakoff et al., 2003).

## 4 Results

### 4.1 Measurement model

Preliminary data screening indicated no substantial violations of the assumptions examined. Across the 57 observed items, skewness ranged from −0.278 to 0.975 and kurtosis from −1.202 to −0.141. The maximum Mahalanobis distance was 90.936, below the critical value of 95.751 (df = 57, *p* = 0.001), and tolerance and VIF values ranged from 0.546 to 0.647 and from 1.546 to 1.831, respectively. Thus, no multivariate outliers or problematic multicollinearity were identified, and no additional observations were excluded. Preliminary reliability analysis was conducted using SPSS. The four-factor measurement model was then tested using CFA in AMOS, with mindfulness, psychological flexibility, self-compassion, and anxiety estimated simultaneously as four latent constructs and their respective scale items specified as indicators. The measurement model is presented in Supplementary Figure S1. The four-factor measurement model showed a good fit to the data (*χ*^2^/df = 1.313, NFI = 0.901, TLI = 0.973, CFI = 0.974, RMSEA = 0.025, SRMR = 0.036) (Hair et al., 2019). The standardized factor loadings for all measurement items were significant and ranged from 0.705 to 0.823 (Table 2). All constructs satisfied the criteria for reliability and validity, with Cronbach’s *α* and composite reliability exceeding 0.90 and average variance extracted (AVE) exceeding 0.50, thus supporting internal consistency and convergent validity (Fornell and Larcker, 1981). Discriminant validity was also supported, as the square root of each construct’s AVE was greater than its correlations with the other constructs.

**Table 2:** Reliability and validity.

Items	Loading	Cronbach’s α	CR	AVE
Mindfulness (M)		0.952	0.953	0.573
M1	0.719			
M2	0.776			
M3	0.794			
M4	0.766			
M5	0.740			
M6	0.735			
M7	0.735			
M8	0.733			
M9	0.778			
M10	0.823			
M11	0.745			
M12	0.814			
M13	0.715			
M14	0.758			
M15	0.715			
Psychological flexibility (PF)		0.966	0.966	0.553
PF1	0.752			
PF2	0.732			
PF3	0.741			
PF4	0.726			
PF5	0.725			
PF6	0.733			
PF7	0.707			
PF8	0.755			
PF9	0.785			
PF10	0.754			
PF11	0.817			
PF12	0.705			
PF13	0.723			
PF14	0.738			
PF15	0.784			
PF16	0.748			
PF17	0.766			
PF18	0.763			
PF19	0.717			
PF20	0.726			
PF21	0.711			
PF22	0.756			
PF23	0.726			
Self-Compassion (SC)		0.942	0.942	0.575
SC1	0.725			
SC2	0.721			
SC3	0.753			
SC4	0.797			
SC5	0.753			
SC6	0.773			
SC7	0.742			
SC8	0.744			
SC9	0.759			
SC10	0.778			
SC11	0.734			
SC12	0.817			
Anxiety (A)		0.909	0.910	0.591
A1	0.773			
A2	0.754			
A3	0.768			
A4	0.742			
A5	0.727			
A6	0.804			
A7	0.808			

### 4.2 Structural model

After validation of the measurement model, the structural model was examined in AMOS 24. The structural model showed a good fit to the data (*χ*^2^/df = 1.311, NFI = 0.901, TLI = 0.973, CFI = 0.974, RMSEA = 0.025, SRMR = 0.0364). Table 3 shows significant correlations among mindfulness, psychological flexibility, self-compassion, and anxiety (*p* < 0.01).

**Table 3:** Pearson correlations.

Variables	Mindfulness	Psychological Flexibility	Self-Compassion	Anxiety
Mindfulness	(0.757)			
Psychological Flexibility	0.581**	(0.744)		
Self-Compassion	0.529**	0.634**	(0.759)	
Anxiety	−0.516**	−0.546**	−0.613**	(0.769)

**p < 0.01. Values in parentheses on the diagonal represent the square roots of AVE.

As shown in Figure 2, mindfulness was significantly positively associated with both psychological flexibility (*β* = 0.583, *p* < 0.001) and self-compassion (*β* = 0.250, *p* < 0.001), supporting Hypotheses 1 and 2. Psychological flexibility was significantly positively associated with self-compassion (*β* = 0.488, *p* < 0.001), supporting Hypothesis 3. Regarding anxiety, both psychological flexibility (*β* = −0.264, *p* < 0.001) and self-compassion (*β* = −0.448, *p* < 0.001) were significantly negatively associated, supporting Hypotheses 4 and 5. The model explained 34% of the variance in psychological flexibility, 44% in self-compassion, and 42% in anxiety.

**Figure 2:**
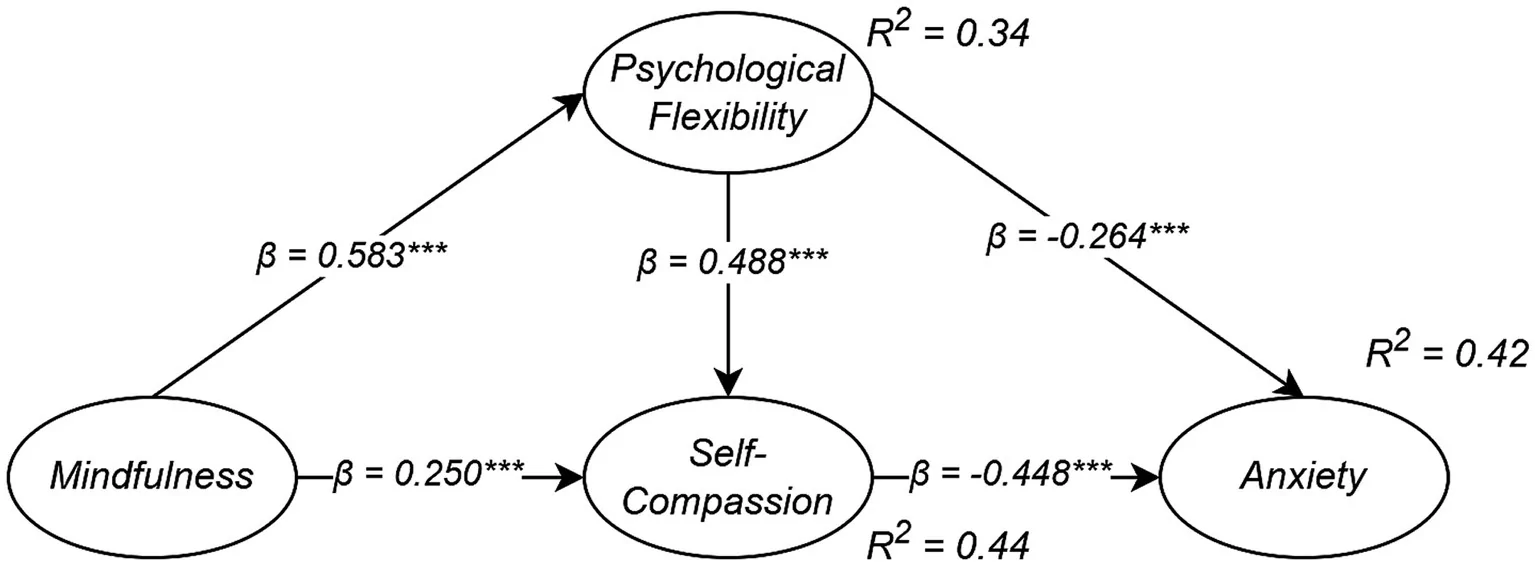
Structural model. ***p < 0.001.

The structural model was specified to examine the hypothesized serial indirect pathway; therefore, the direct path from mindfulness to anxiety was not included. The hypothesized serial indirect effect was examined using 5,000 bootstrap resamples with bias-corrected 95% confidence intervals (Table 4). The serial indirect effect from mindfulness to anxiety through psychological flexibility followed by self-compassion was significant (*β* = −0.128, *p* < 0.001, 95% CI [−0.167, −0.096]), supporting Hypothesis 6.

**Table 4:** Structural and serial indirect effects.

Hypothesis	Path	*β*	*SE*	*p*	95% CI
H1	M → PF	0.583	0.033	0.001	[0.513, 0.643]
H2	M → SC	0.250	0.046	< 0.001	[0.157, 0.337]
H3	PF → SC	0.488	0.039	< 0.001	[0.410, 0.564]
H4	PF → A	−0.264	0.052	< 0.001	[−0.365, −0.157]
H5	SC → A	−0.448	0.048	< 0.001	[−0.543, −0.355]
H6	M → PF → SC → A	−0.128	0.018	< 0.001	[−0.167, −0.096]

M = Mindfulness; PF = Psychological Flexibility; SC = Self-Compassion; A = Anxiety. The 95% confidence intervals are bias-corrected bootstrap confidence intervals based on 5,000 resamples.

## 5 Discussion

### 5.1 Theoretical contributions

This study contributes to the literature on young family caregivers by clarifying a psychological pathway through which mindfulness is associated with anxiety. Previous research has shown that young carers and young adult caregivers are at elevated risk of psychological distress, but relatively less attention has been given to the internal psychological processes that may explain why some young caregivers experience lower anxiety than others under caregiving stress. The present findings suggest that mindfulness is indirectly associated with anxiety through psychological flexibility and self-compassion, two adaptive resources that may be especially important when young people must manage family care while also facing developmental demands.

First, among young family caregivers, both mindfulness and psychological flexibility were significantly positively associated with self-compassion, and mindfulness was also significantly positively associated with psychological flexibility. This finding aligns with previous research (Böge et al., 2023; Hollis-Walker and Colosimo, 2011; Marshall and Brockman, 2016). That is, mindfulness may be associated with differences in the way young family caregivers process stress, and with a lower tendency to become overly preoccupied with threatening information when confronted with the responsibilities, role conflicts, and future uncertainties associated with long-term caregiving, thereby demonstrating higher levels of psychological flexibility (Böge et al., 2023). Greater psychological flexibility may be associated with facing the fatigue, limitations, and emotional fluctuations inherent in the caregiving process with greater openness rather than defensiveness. In turn, this process may be associated with less self-criticism about personal shortcomings and a more compassionate and accepting view of oneself, consistent with greater self-compassion (Marshall and Brockman, 2016). Young family caregivers often experience long-term stress. In this group, mindfulness, psychological flexibility, and self-compassion should not be treated as separate protective factors. Instead, they may form an interconnected system of psychological resources. This system may be associated with psychological adaptation in the context of long-term caregiving.

Second, anxiety among young family caregivers was significantly negatively associated with both psychological flexibility and self-compassion. This finding aligns with previous research (MacBeth and Gumley, 2012; Van Dam et al., 2011; Yao et al., 2023). For young family caregivers, although both of these psychological resources may help mitigate anxiety, they appear to operate at different levels. Psychological flexibility is primarily related to how individuals process stress and may be associated with lower experiential avoidance and fewer catastrophic interpretations when confronted with caregiving burdens, role conflicts, and uncertainty about the future, which may be associated with lower anxiety (Tan et al., 2023). Self-compassion primarily shapes how individuals respond to themselves and may be associated with lower levels of self-blame, self-denial, and psychological exhaustion when experiencing fatigue, helplessness, frustration, or emotional fluctuations (Wiita et al., 2024). For young people who are at a critical developmental stage yet have prematurely assumed family caregiving responsibilities, anxiety arises not only from external pressures themselves, but is also closely related to how they interpret these pressures and treat themselves.

The present study also identified a significant serial indirect association between mindfulness and anxiety through psychological flexibility and self-compassion. Previous research has linked young caregiving with poorer mental health, lower well-being, and greater perceived stress (Fleitas Alfonzo et al., 2024; Greene et al., 2017; Wepf and Leu, 2022). The present findings suggest that these psychological difficulties may also be understood in relation to the way internal regulatory resources are interconnected. Higher mindfulness was associated with greater psychological flexibility, which was in turn associated with greater self-compassion, while greater self-compassion was associated with lower anxiety. This pattern is consistent with the distinct but related functions of psychological flexibility and self-compassion. Psychological flexibility reflects the capacity to remain open to difficult internal experiences and respond to them adaptively, whereas self-compassion involves responding to personal distress and limitations with greater understanding and acceptance (Fledderus et al., 2013; Marshall and Brockman, 2016). For young family caregivers, this distinction is particularly relevant because caregiving-related stress involves not only managing difficult experiences, but also responding to personal fatigue, limitations, and perceived inadequacy. The observed sequence therefore suggests that flexible engagement with stressful experiences and a more compassionate response to the self may represent connected aspects of psychological adjustment in this population.

Drawing on the stress process model, this study highlights the importance of internal psychological resources in relation to anxiety among young family caregivers. It also provides a clearer view of how these resources work. Within this framework, psychological outcomes are not determined by stressors alone. They arise from the combined effects of primary and secondary stressors, the psychosocial resources available to the individual, and the coping processes used to deal with stress (Pearlin et al., 1981). Among young family caregivers, the present findings suggest that internal resources are not best understood as separate protective factors operating side by side. Instead, these resources were associated in the theoretically specified sequence and were jointly related to anxiety in young family caregivers. Psychological flexibility reflects the capacity to process caregiving stress and regulate responses to it (Han et al., 2025). Self-compassion, by contrast, reflects the ability to respond to one’s own distress with understanding and acceptance (Leary et al., 2007). From this perspective, anxiety in young family caregivers is not only associated with caregiving demands. It also reflects how stress is experienced and how internal psychological resources are brought into use. This interpretation aligns with the stress process model. In that model, psychological outcomes are shaped through the combined effects of stressors, available resources, and coping processes. It also fits the resource-buffering view of stress (Haley et al., 1987; Pearlin et al., 1981). On this basis, efforts to understand and address anxiety in young family caregivers should not be limited to reducing external demands or relieving immediate distress. Equal attention should be given to the development of stress regulation and self-regulation capacities, as well as to the gradual strengthening of internal psychological resources.

### 5.2 Practical implications

These findings suggest several potential directions for supporting young family caregivers experiencing anxiety. In this population, anxiety appears to be associated not only with caregiving demands, but also with the ways stressful experiences are understood and managed. Accordingly, practical support may consider both reducing caregiving strain and strengthening psychological resources related to adaptation. Such support may involve schools, community services, families, and young caregivers themselves.

In school and service settings, early recognition and ongoing support are both important. Educational and counseling staff need to pay attention to signs such as persistent fatigue, a clear decline or instability in academic performance, frequent absences, and continuing caregiving responsibilities. When these risks become evident, schools may need to provide more flexibility in coursework, attendance requirements, and access to psychological support. Community and social service agencies may provide additional support by offering accessible information on care resources, opportunities for peer connection, and timely mental health services. Assessment should also consider the family caregiving context, so that psychological support can be connected with family assistance and practical care resources.

Within the family, caregiving responsibilities should not fall mainly on the young person, especially when those demands go beyond what is developmentally appropriate. Families may need to reconsider how care tasks are shared, make expectations clearer, and create more open communication. This may allow young caregivers to express fatigue, personal limits, and support needs more openly. When necessary, families can also turn to community care, psychological services, and other forms of external support to ease excessive demands.

At the individual level, mindfulness, psychological flexibility, and self-compassion may represent potential targets for future intervention development. Mindfulness-based practices could be explored as a way of increasing awareness of stress reactions and difficult emotions, while approaches targeting psychological flexibility may be relevant to reducing rigid responses to caregiving demands and role conflict. Self-compassion-based strategies may likewise be considered for addressing self-blame and harsh self-criticism during periods of fatigue, helplessness, or emotional distress.

Overall, these findings point to the potential value of considering support at multiple levels, including systemic support, adjustments within the family, and psychological resources at the individual level. Because the present study was cross-sectional and did not evaluate intervention effects, future intervention studies are needed to determine whether these approaches are effective in reducing anxiety and supporting adaptation among young family caregivers.

### 5.3 Limitations

(1) Due to the cross-sectional design, causal direction and temporal ordering among the variables cannot be established. Future longitudinal studies are needed to examine the dynamic processes through which these variables influence one another over time.

(2) Because participants were recruited using non-probability convenience sampling from three cities in Hunan Province, the sample may not fully represent the broader population of young family caregivers. This may limit the representativeness and generalizability of the findings. Future research should therefore examine young family caregivers from a wider range of regions, cultural backgrounds, and demographic groups.

(3) The caregiving context was captured only in a limited way in the current study, and detailed information on caregiving intensity, duration, and specific caregiving activities was not collected. Future research should include more detailed assessments of caregiving intensity, caregiving duration, and care-recipient characteristics to better understand how different caregiving circumstances are associated with anxiety among young family caregivers.

(4) All focal variables were assessed using self-report measures, which may be subject to response bias and common-method variance. Although Harman’s single-factor test did not indicate a serious common-method bias, this possibility cannot be fully excluded. Future studies could incorporate multiple methods or data sources to reduce reliance on self-report measures.

## 6 Conclusion

The study met its objectives and supports the relevance of the stress process model to young family caregiving. Psychological flexibility and self-compassion were both associated with lower anxiety, and a significant serial indirect association was observed between mindfulness and anxiety through psychological flexibility followed by self-compassion. Anxiety in young family caregivers may be understood in relation to internal psychological resources associated with how stressful experiences are processed and how young caregivers respond to their own distress. Within the proposed model, greater psychological flexibility was associated with greater self-compassion, and greater self-compassion was associated with lower anxiety.

These findings point to the potential value of considering both psychological resources and external support when addressing the needs of young family caregivers. Schools and community services may consider strengthening early identification and providing continuous support. Families may also consider more balanced sharing of caregiving responsibilities and more open communication. At the individual level, mindfulness, psychological flexibility, and self-compassion may represent potential targets for future intervention development. The effectiveness of these approaches in reducing anxiety and supporting adaptation among young family caregivers should be evaluated in future intervention studies.

## Data Availability

The datasets supporting the findings of this study are included in the Supplementary material. Further inquiries can be directed to the corresponding author.
